# 2D framework materials for energy applications

**DOI:** 10.1039/d0sc05889k

**Published:** 2020-12-23

**Authors:** Andreas Schneemann, Renhao Dong, Friedrich Schwotzer, Haixia Zhong, Irena Senkovska, Xinliang Feng, Stefan Kaskel

**Affiliations:** Department of Inorganic Chemistry, Technische Universität Dresden Bergstr. 66 01069 Dresden Germany Andreas.Schneemann@tu-dresden.de; Center for Advancing Electronics Dresden (CFAED), Faculty of Chemistry and Food Chemistry, Technische Universität Dresden 01062 Dresden Germany

## Abstract

In recent years a massive increase in publications on conventional 2D materials (graphene, h-BN, MoS_2_) is documented, accompanied by the transfer of the 2D concept to porous (crystalline) materials, such as ordered 2D layered polymers, covalent-organic frameworks, and metal–organic frameworks. Over the years, the 3D frameworks have gained a lot of attention for use in applications, ranging from electronic devices to catalysis, and from information to separation technologies, mostly due to the modular construction concept and exceptionally high porosity. A key challenge lies in the implementation of these materials into devices arising from the deliberate manipulation of properties upon delamination of their layered counterparts, including an increase in surface area, higher diffusivity, better access to surface sites and a change in the band structure. Within this minireview, we would like to highlight recent achievements in the synthesis of 2D framework materials and their advantages for certain applications, and give some future perspectives.

## Abstract 2

In recent years the 2D concept has been transferred from conventional 2D materials to porous 2D framework materials. This minireview takes a closer look onto the preparation of 2D framework materials and their merits for energy applications.

## Graphical abstract

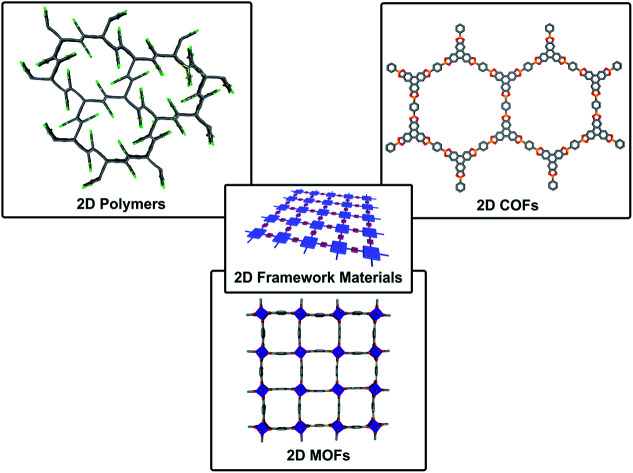

## Introduction

The discovery of graphene and the determination of the strikingly different properties compared to its three-dimensional bulk analogue graphite spawned tremendous research efforts among different scientific communities.^1,2^ First, graphene and graphene-like materials (*i.e.* reduced graphene oxide (rGO), graphyne, fluorographene) were at the center of attention,^3^ but soon other two-dimensional materials (2DMs) followed, such as hexagonal boron nitride (h-BN),^4^ 2D dichalcogenides (*i.e.* MoS_2_, WSe_2_),^6,7^ 2D oxides,^8^ MXenes (*i.e.* Ti_2_C),^9^ black phosphorus^5^ and many more. 2DMs can be generally defined as materials with infinite crystalline extensions along two dimensions and one crystalline dimension with few or single atomic layers thickness. Essentially, 2DMs can be derived from most classes of known layered materials, which possess strong in-plane bonds within the layers and only weak interactions between neighbouring layers. It can be expected, that 2D framework materials combine the versatile properties of bulk and the unique features of two-dimensional matter. The resulting properties may range from unique electronic and optical properties caused by bandgap shifting, to quantum confinement of electrons, excitons and phonons in the two dimensional layers, high mechanical strength, an increase in accessibility of surface sites and faster diffusion through the material, making 2DMs excellent candidates for optoelectronic and energy storage related applications. Apart from these “conventional” 2DMs, also an increased interest has risen in 2D framework materials (2DFMs). We want to define them here as 2DMs synthetically constructed from molecular building blocks, featuring (crystalline) short and long-range order, as well as accessible, regular in-plane pores. This includes the classes of 2D ordered polymers (2DPs),^10^ 2D covalent-organic frameworks (2D COFs)^11^ and 2D metal–organic frameworks (2D MOFs),^12^ while excluding classical polymers and similar materials (*i.e.* 2D conjugated microporous polymers,^13^ porous aromatic frameworks^14^) that do not possess mentioned crystalline order and narrow pore-size distribution. Further we also exclude non-porous coordination polymers.

**Fig. 1:**
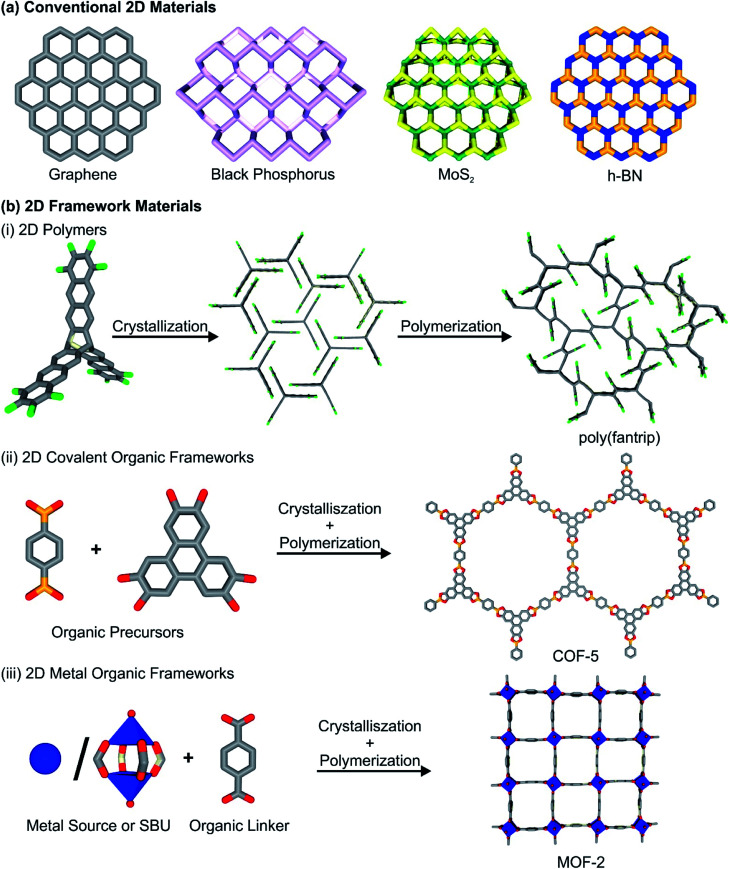
(a) Schematic depiction of a range of conventional 2D materials. (b) 2D framework materials, their building blocks and synthetic procedures: (i) 2D polymers, (ii) 2D covalent-organic frameworks and (iii) 2D metal–organic frameworks.

2D COFs and 2DPs are synthesized by the reaction of organic, commonly aromatic multitopic monomers through strong covalent bonds, creating a porous, crystalline framework structure. In some cases, the terms 2D COFs and 2DPs are used synonymously throughout the literature, and the distinction between these two classes of materials is rather blurry. We want to follow a differentiation between the two classes of materials, as already stated elsewhere (see Fig. 1 for construction principles of different 2DFMs).^15^ 2D layered COFs and 2DPs are distinguished in here by the synthetic procedures they are derived by. While for 2D layered COFs, polymerization and crystallization occur simultaneously during the synthesis, in the case of 2DPs crystallization and polymerization are decoupled. In a first step the monomers of the 2DPs are assembled in a crystalline fashion, arranging the connection points in a close manner and in a second step a single-crystal to single-crystal transformation occurs during polymerization of the components. We think that this sharp distinction is necessary to describe the underlying properties, as 2D polymers are distinctively more crystalline than COFs, but the number of reported frameworks is significantly lower due to the limited number of suitable precursors and reactions in comparison to the larger variety of organic reactions used to access COFs. In contrast to this, 2D MOFs are composed of organic multitopic monomers, which are linked through coordination bonds with metal-containing secondary building units (either metal ions or metal-oxo clusters). The formed bonds between the building blocks are considerably weaker than in the case of 2D COFs and 2DPs, but their dynamic character allows for higher crystallinity than in the related 2D COF structures. Also, we feel it is necessary to state that in the scientific literature the term 2D COF and 2D MOF are often used to describe 2D layered structures, essentially 3D materials. Throughout the review, however, the term 2D COF and 2D MOF will be reserved for thin, single or few layer versions of the frameworks, while we define the non-exfoliated precursor materials as layered COFs and layered MOFs herein. We highly urge the community to discuss the current nomenclature, and recommend to add the word “layered” to discuss 3D frameworks featuring strong bonds (*i.e.* covalent, coordination bonds) along two dimensions and weak bonds (hydrogen bonds, π–π interactions) along the third dimensions. Furthermore, 2D thin films of COFs, MOFs and 2D polymers will also not be discussed in detail in this review, even though they are technically speaking 2DFMs, but elsewhere they have been discussed and reviewed in much detail.^16–19^

**Fig. 2:**
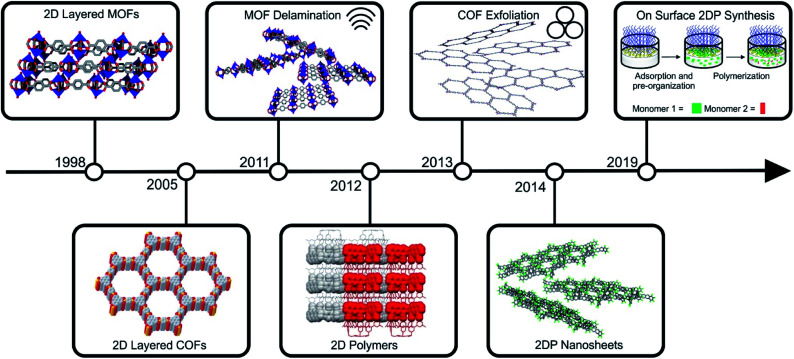
Timeline of important milestones in the synthesis of layered framework materials and the preparation of their 2D counterparts. Parts of this figure have been reproduced from ref. 22, 33 and 38. Copyright © 2005, American Association for the Advancement of Science. Copyright © 2012 and 2019 Nature Publishing Group.

Notably, during the early stages of MOF and COF research, layered framework materials, were among the early pertinent examples reported in the literature (for instance Cu_2_(bdc)_2_ and COF-1).^21,22^ The development of their two-dimensional analogues followed soon after the discovery of graphene (see Fig. 2 for a timeline). Arguably, the first targeted 2D MOFs were MOF-2 (Zn_2_(bdc)_2_(H_2_O)_2_) and the related Cu_2_(bdc)_2_(X)_2_ (X = coordinated solvent).^23,24^ These structures and their derivatives remain among the most investigated compounds within this class of materials, and their utility for energy related application was highlighted through their employment as a filler material for CO_2_/CH_4_ separation membranes.^25^ The first reports on the exfoliation of 2D layered COFs were published by Banerjee and co-workers, who introduced the use of mechanochemistry to delaminate imine-based layered COFs.^26^ Conjugated COF systems have been of high interest in the literature for the use in optoelectronic and energy storage devices and their nanoscaling could even enhance these properties.^20,27–32^ The development of 2D polymers is, in comparison to 2D layered COFs and MOFs, still in its infancy, due to the difficult realization of the precursor molecules and the arguably more straight forward preparation of 2D layered COFs and MOFs. The first example of a 2D polymer was published in 2012 by Kissel *et al.*^33^ and in 2014 the first example of a porous 2DP followed, which could be readily exfoliated into monolayers.^34,35^ Recently, interfacial synthesis has proven to be a reliable tool to produce ultrathin 2DPs with large lateral extensions.^36–38^

Currently, the field of 2DFMs shows a lot of advancements. Rapid improvements in their synthesis and properties are made, and growing interest towards energy related applications is observed.^39^ Evidently, many potential applications make good use of the enhanced diffusion pathways created by the downsizing along one dimension of the crystal structures and the resulting improved access to catalytic or redox active sites, which are important features for ion storage in supercapacitors or for faster redox reactions in batteries and during electrocatalysis. Further, novel optical properties arise through the formation of 2D arrays of fluorophores and the tuning of the materials bandgap through nanosizing. Additionally, the permeability for gases is enhanced while retaining selectivity in separation technologies. Within this minireview, we highlight recent advances in the preparation of 2D framework materials, discussing accessible structures, achievable thicknesses and lateral extensions, and their implications for energy applications.

## Materials

### 2D polymers

As an emerging class of 2DFMs, 2DPs comprise single-atom/monomer-thick, covalent-bonded networks with well-defined periodicity along two orthogonal directions,^40,41^ which have broad application scope in separation, catalysis, optoelectronics, sensing and energy storage and conversion.^10^ However, no real structurally-defined 2D polymer has been obtained until the discovery of graphene, an archetypical example from nature.^1^ Over the last decade, vigorous efforts have been devoted to the rational synthesis of 2DPs. Typically, top-down exfoliation of synthetic laminar structures, has been successfully employed to achieve 2DPs.^34,35,42^ The lateral sizes of the obtained 2DP sheets rely on the size of the single crystals, and the precise thickness control and unambiguous structural definition of the exfoliated nanosheets requires additional effort.^43^ Bottom-up on-surface synthesis has also enabled the preparation of various 2DP networks under ultrahigh vacuum condition.^44–47^ Those strategies are generally restricted in terms of lateral size, small crystalline domains (typically tens of nanometers) and high defect density, due to the low mobility of monomers and reactivity at the solid–vacuum interfaces. Moreover, a transfer of such metal surface-bound polymer networks for fundamental studies is complicated.

As another bottom-up strategy, air–water and liquid–liquid interface assisted synthesis have recently been explored towards the construction of 2DPs.^48,49^ Such interfacial methods offer the possibility to overcome the limited diffusion rate of monomers, leading to macroscopic 2DPs for which the interface acts as the template for the confined polymerization of monomers into 2D. For instance, Feng *et al.* demonstrated the synthesis of imine-based 2DPs at the air–water and liquid–liquid interfaces,^50^ which are free-standing, single- and few-layer polycrystalline films respectively. Nevertheless, the crystallinity, *i.e.*, degree of long-range order of the covalent-bonded repeating units of the resultant 2DPs remains unsatisfactory with small crystalline domain sizes of ∼10–20 nm. The synthesis of highly crystalline 2DPs requires the development of new interfacial synthesis strategies and addressing of the synthetic mechanism, grain size, grain boundaries and edge structures remains under development.

**Fig. 3:**
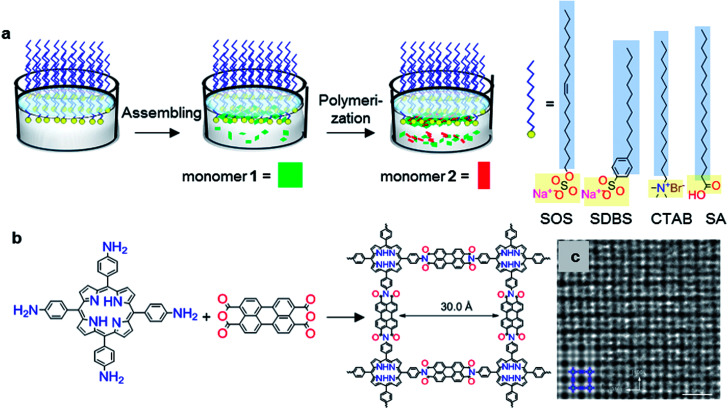
Synthesis of structurally-ordered 2D polyimide crystal using a novel air–water interface method assisted by surfactant monolayer: (a) synthesis procedures, (b) and reaction scheme with precursors; (c) HRTEM image of resultant 2D polyimide crystals. Scale bar: 5 nm. Adapted from ref. 38 with permission. Copyright© 2019, the author(s), under exclusive licence to Springer Nature Limited.

Very recently, a surfactant-monolayer-assisted interfacial synthesis (SMAIS) method has been developed for the preparation of few-layer 2D polyimide and 2D polyamide crystals on the water surface (Fig. 3a),^38^ through reaction between amine and anhydride monomers. The surfactant layer can guide the supramolecular self-assembly of monomers on its hydrophilic side *via* weak interactions (such as hydrogen bonds, electrostatic interactions, and van der Waals forces), and further provides a 2D confinement geometry for the polymerization into 2D. Based on the SMAIS method, the synthetic 2D polyimide films (Fig. 3b) exhibited a large area with lateral size up to several cm^2^, a thickness of approximately 2 nm (corresponding to ∼5 layers), and an average crystal domain size of around 3.5 μm^2^. The 3 nm lattice of 2DPI can be clearly revealed by selected area electron diffraction (SAED) and high-resolution TEM (HRTEM) imaging (Fig. 3c). Nevertheless, the crystalline area (*vs.* amorphous region) is below 70% of the whole film. This SMAIS strategy was further extended to the polycondensation reaction of an amine functionalised porphyrin monomer and 1*H*,3*H*-furo[3,4-*f*][2]benzofuran-1,3,5,7-tetrone, providing a crystalline few-layer 2D polyamide with dual-pore lattice structures. Under the SOS monolayer, 2D polyamide adopted a face-on configuration with a crystal domain size of ∼0.3 μm^2^. By utilizing an octadecanoic acid (stearic acid, SA) monolayer, edge-on-oriented 2D polyamide could be achieved with a significantly increased domain size (∼121 μm^2^). This result indicated that the surfactant, depending on its polar head, promoted the arrangement of the monomers—and in turn their polymerization—either horizontally or vertically with respect to the water surface, which is also helpful for the controlled synthesis with directional layer orientation. Currently, the SMAIS method has also been successfully utilized to synthesize highly crystalline quasi-2D polyaniline films,^51^ 2D polyimine films,^52,53^ and boronate ester 2D COF films.^54^ Benefiting from the high crystallinity and thin-film processability with controlled film thickness as well as the intrinsic (semi)conducting nature, these 2DPs have been incorporated into thin-film sensor, logic and memory devices.

### 2D covalent organic frameworks

Arguably, among the archetypical covalent-organic framework structures, many of the most prominent examples are actually layered structures.^22,55,56^ Even though also extensive research has been put into the development of 3D COFs.^57,58^ Commonly, layered COFs are constructed from simple organic building blocks, for instance through the trimerization of ditopic linear linkers or through the combination of trigonal or tetragonal linkers with linear building blocks. Reactions typically used to fuse the building blocks are condensation reactions to form boroxines^59^ and triazines,^60^ Schiff base reactions, esterification between diols and boronic acids,^61^ and many more.^62–68^ Recently also multicomponent reactions^69,70^ have emerged to fuse these structures together, creating more stable bonding units between the building blocks. A common problem among 2D layered COFs – low crystallinity – arises from the nature of their strong, non-dynamic, covalent bonding, and hence the production of large area 2D COFs through exfoliation of layered COFs is hampered. Nonetheless, a lot of effort has been made to develop approaches to improve the crystallinity of layered COFs.^71,72^

The adjacent layers in COFs adhere to each other through π–π interactions or even by hydrogen bonds.^73^ A number of methods has been developed to break these interactions and obtain nanosheets of layered COFs (2D COFs). Among them are the intercalation with different liquid additives, solvents, or ions; the targeted incorporation of ionic groups^74^ or bulky side chains; and mechanochemical approaches.^26^ Additionally, there are also some bottom up methods such as interfacial growth (liquid–liquid, liquid–solid interface) or rapid exchange synthesis. In the following, we discuss a number of methods and the resulting materials with respect to their thickness and lateral dimensions. Arguably, COF thin films are also 2D materials,^75^ but we want to limit our perspective to freestanding 2D materials. Notably, a large body of literature exists on the preparation of nanosheets based on the imine bond created by the reaction of amines with aldehydes, most likely because the synthesis of the respective bulk 2D layered COFs is well explored and the materials are quite chemically robust. However, also some marked examples on the delamination of triazine and boronic acid based COFs exist.

**Fig. 4:**
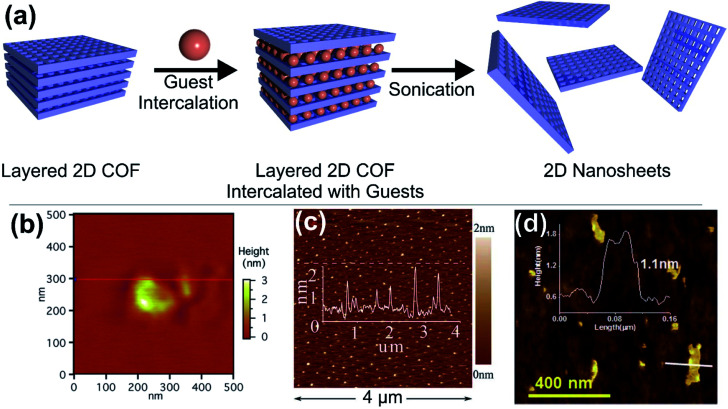
(a) Schematic illustration of the solvent assisted delamination of layered COFs. (b–d) Atomic force micrographs of (b) hydrazone bridged COF-43 exfoliated in the presence of dioxane, (c) triazine bridged CTF-1 in the presence of piranha solution and (d) an imine bridged COF exfoliated from water. Reproduced from ref. 77 with permission from the American Chemical Society and from ref. 78 and 79 with permission from the Royal Society.

Among the methods to obtain 2D COFs, the solvent assisted intercalation of layered COFs is prevalent (like for many conventional 2D materials, Fig. 4a). Generally, polar solvents such as H_2_O and short chain alcohols are used and in the case of some robust COFs also acids can be utilised.^76^ However, there are also reports on the use of acetonitrile or dioxane. In one of the first examples Bunck *et al.* were able to exfoliate hydrazone linked layered COFs *via* solvent intercalation, for instance with dioxane and water.^77^ After delamination platelets with lateral extensions of 200 nm were obtained in the case of dioxane, with an average thickness of 1.32 nm (Fig. 4b), corresponding to 3–5 layers. In the case of H_2_O, even bi- or single layers could be obtained. Other examples include the use of water by Das *et al.,* achieving thicknesses as low as 1 nm for imine based COFs^78^ (Fig. 4d) and the use of piranha solution by Zhu *et al.*, which yielded 0.7 nm thick sheets of triazine based COFs, however this rather harsh treatment also reduced the lateral extensions to 35 nm or less (Fig. 4c).^79^

The delamination of layered COFs through mechanical force was first established by the group of Banerjee in 2013.^26,80^ In their work, differently functionalized imine-based COFs were ground by mortar and pestle to obtain COF nanosheets with thicknesses of 3–10 nm. Lei and co-workers were able to exfoliate an imine based COF by mechanical polishing for 8 hours followed by ball-milling in ethanol for 30 minutes.^81^ The procedure was performed in the presence of CNTs and structures which are on average thinner than 2 nm could be achieved. Wang and co-workers prepared a series of different imine-based COFs, which were all exfoliated *via* ball milling.^82^ For this series 3–5 nm thick sheets could be produced, which corresponds to 10–15 structural units (layers). Sheets with micron-sized lateral extensions could be prepared by Zhang and colleagues.^83^ A perfluorinated analogue of the triazine-based framework CTF-1 was prepared through ionothermal synthesis. Ball milling for 10 hours yielded large sheets, with 2–3 μm in size and a thickness of only 4 nm.

An interesting approach was shown by the group of Banerjee, through the post-synthetic modification of imine-based COFs using linkers with an anthracene backbone.^84^ The anthracene moiety can undergo a Diels–Alder reaction with *N*-hexylmaleimide. Addition of this bulky side groups interferes with the π–π stacking of adjacent layers and leads to the exfoliation of the bulk, layered structure, generating nanosheets with a thickness of 17 nm and lateral extensions of 500 × 200 nm. Building up on this work, Haldar *et al.* were able to react a similar anthracene based imine-linked COF with maleic anhydride, creating 1–1.5 nm thick 2D COFs (2–5 layers) with large extensions of up to 3 μm after solvent assisted exfoliation.^85^ Although this approach seems very promising, it is somewhat limited to the use with anthracene based frameworks and is not universally applicable.

Another methodology to weaken the interaction between adjacent layers is the incorporation of ionic groups on the linker backbone of covalent organic frameworks. This strategy is also sometimes referred to as the surface charge regulation approach, which in some cases allows for exfoliation and spontaneous reassembly upon a stimulus. An example for such a COF is the imine-based material EB-COF,^86^ which features an ethidium bromide derived linker molecule. This material was exploited by Mal *et al.* for exfoliation and the charged layers readily exfoliated in deionized water into 1.5 nm thick sheets, which corresponds to two layers of the COF material.^87^ In a follow up study the related propidium iodide cationic COF was prepared and it readily self-exfoliated in water to sheets with an average thickness of 1.6 nm.^88^ Interestingly, this material can be restacked by the use of a macrocyclic host, in this case cucurbit[7]uril, through complexation of the quaternary ammonium groups on the propidium side groups. Addition of 1-adamantylamine hydrochloride leads to decomplexation and the material exfoliates again.

The use of interfacial synthesis to generate 2D sheets of covalent-organic frameworks is still used scarcely. In contrast to the previously mentioned top-down approaches, this represents a bottom-up approach, where the building blocks of the COF are assembled at an interface, for instance a liquid–liquid or liquid solid interface. For instance, Shi *et al.* prepared a series of imine-linked COFs through the interface confined growth of the nanosheets on the surface of table salt crystals.^89^ For the three materials they grew, nano-sheets with thicknesses of 3.2–4.8 nm and large lateral extensions of 1–4 μm were obtained. Ma and co-workers showed the interfacial growth of an imine based COF at a liquid–liquid interface.^90^ The COF is build up by the condensation of an aldehyde and an amine. The aldehyde is dissolved in DCM (dichloromethane) and the imine is dissolved in a mixture of DCM and DMF (*N*,*N*-dimethylformamide). First, the aldehyde solution is placed in the reaction vessel, subsequently acetic acid is carefully layered on this solution and afterwards the imine solution is layered on top. The imine is then slowly diffusing through the acetic acid solution and the COF forms as thin sheets at the DCM–DCM/DMF interface.

In general, it needs to be noted that a lot of research is focused on derivatives of a few systems, particularly for imine-based COFs many strategies have been developed. We believe that moving away from imine-based systems and looking at isoreticular structures with different organic linkages would be a beneficial step to develop a better understanding of the processes involved in COF delamination. It is somewhat obvious, that the π–π interactions between the linkers of neighbouring layers are responsible for their adhesion. Hence, disturbing these interactions is a helpful strategy to facilitate exfoliation, however at the same time the property giving units of the COF are also located on those linker backbones. Finding ways that lead to repulsion between neighbouring linkage units might be a beneficial step to improve exfoliation while preserving linker-derived properties of the framework. Furthermore, there is also some room left for improvement concerning the lateral extensions of 2D COFs (*i.e.* most obtained large area sheets are comparatively thick), which in many cases are also not properly reported. Probably the improvement of the crystallinity of parent layered structures will give rise to larger 2D sheets after exfoliation and the use of COF structures with stronger in-plane bonds might inhibit bond breaking along the plane during harsher exfoliation approaches.

### 2D metal–organic frameworks

Metal–organic frameworks are porous, crystalline solids^91^ built-up from metal centers^92^ bridged by organic linkers^93^*via* coordination bonds and the majority of them are 3D networks, even though a plethora of layered and pillared-layer structures is known as well.^94–96^ The diversity of metal coordination modes and the versatility of organic linker molecules allow MOFs to develop regularly arranged pores with tailored pore sizes and functionality, opening up an immense potential for applications in many different technological fields.^97,98^ Layered MOFs are expected to have lower porosity and higher network densities in comparison to their 3D counterparts. However, the isolation of single, atomically thin MOF layers (sometimes referred to as MOFenes) would enable an in-depth investigation of surface phenomena, chemical reactivity, change in band structure *etc.* Since nanosheets, with regularly repeating metal sites and organic moieties are expected to exhibit unique physical and chemical properties, they are considered as promising nanomaterials for various applications such as separation, energy storage, sensing or catalysis.

**Fig. 5:**
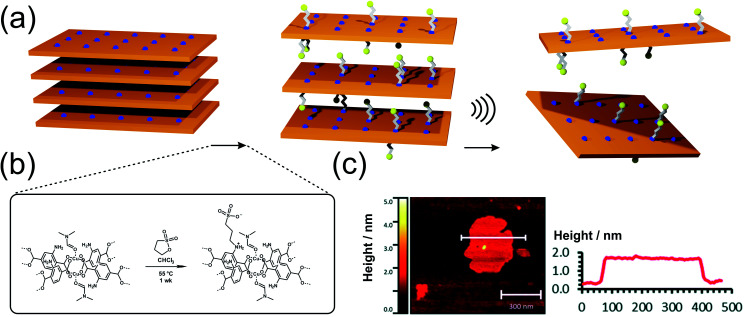
(a) Schematic depiction of the postsynthetic anchoring of charged sulfonate groups on the backbone of Cu_2_(NH_2_-bdc)_2_(DMF)_2_ in order to facilitate the exfoliation of the layered structure. (b and c) AFM and height profile of the exfoliated layers. Reproduced from ref. 114 with permission from the Royal Society.

However, the synthesis of 2D MOFs is anything but trivial. As already known from other material classes, 2D MOFs tend to stack due to strong interlayer interactions such as van der Waals forces, π–π stacking and/or hydrogen bonding.^99^ In order to overcome these interactions and create 2D MOFs with layer thicknesses from the atomic up to the ∼10 nm scale, different approaches have been developed.^100,101^ The conventional and often preferred approach, in which layers are separated from the bulk material, is called top-down process. With this method, 2D-nanosheets can be easily generated from well-established MOFs and their derivatives such as M_2_(bdc)_2_ (also known as MOF-2; M^2+^ = Cu^2+^, Zn^2+^, Co^2+^, Ni^2+^)^23,102,103^ with a simple energy input, *e.g. via* ultrasound or in combination with appropriate surfactants.^37,104,105^ However, not only the addition of suitable reagents to reduce the interactions between the individual layers, but also the functionalization of the linker molecules (*e.g.* with alkoxy substituents), or the medium in which exfoliation takes place, can lead to improved exfoliation result.^106–110^ In this way, Liu *et al.* were able to achieve excellent exfoliation yields of Zn_2_(bim)_4_ (bim^−^ = benzimidazolate) of up to 47% (4–6 nm thickness) by using ionic liquids instead of typical delamination solvents such as pure water, methanol, ethanol, acetone, THF or DMF.^108^ Besides the ultrasonic induced delamination of rather simple layered MOFs, nanosheets of larger layered structures can also be exfoliated through mechanical exfoliation methods (*i.e.* ball mill, grinding, cleaving, freeze thaw cycles, shearing forces^111^), however these harsh conditions often lack precision and control of the resulting products, leading to non-uniform thickness and particle size distributions. MOFenes can not only be generated by physical energy input, but also by the smart choice of intercalation agents which weaken the interlayer interactions. Ding *et al.* were able to use a pillar containing disulfide bonds, which was selectively cleaved, thus accessing 2D MOF sheets with thicknesses of 1 nm, which corresponds to a single layer.^112^ In another study by the same group, it was shown that it is possible to replace the ditopic pillar in a 3D pillared-layered framework by a stronger coordinating monofunctional molecule,^113^ to perform surfactant-assisted synthesis combined with post-synthetic delamination steps. It is also possible to use post-synthetic linker functionalization to facilitate exfoliation of layered MOFs, as shown by the group of Foster (Fig. 5). In the MOF-2 analogue, Cu_2_(NH_2_-bdc)_2_, the amino functionality has been converted post synthetically to a sulfonate *via* reaction with 1,3-propanesultone to increase the interlayer spacing and hence to enable delamination.^114^ The post-synthetic reaction step also introduced positively and negatively charged moieties on the sidechains, which also may help in the repulsion of adjacent layers, finally resulting in 1.4 nm thick sheets, which represents monolayers. In comparison, exfoliation of untreated Cu_2_(NH_2_-bdc)_2_ yielded nanosheets with a large thickness distribution and an average thickness of 25 nm only.

In contrast to the top-down methods, bottom-up methods follow the chemically more sophisticated routes of direct synthesizing ultrathin 2D MOFs from metal and linker solutions. The key step here is to selectively restrict crystal growth in one dimension by limiting stacking without affecting the growth in the other two directions.

One of the most widely used bottom-up strategies to create MOFenes is the interfacial synthesis. The use of interfaces (for instance liquid/liquid, liquid/air or liquid/solid at which the reactions between metal node and ligand can occur in a spatially confined manner) causes the formation of a two-dimensional layer at the interface, which results in ultrathin or even single-layer 2D MOFs.^37,115–118^ However, there are also some more interesting approaches for the direct synthesis of extremely thin structures in addition to the methods of interfacial synthesis/Langmuir–Blodget procedures, which are already very well established in the literature. A very efficient and, most importantly, continuous bottom-up synthesis strategy for the preparation of ultrathin MOF nanosheets is using a microdroplet flow reactor. By this approach the lamellar stacking of nanolayers under dynamic growth conditions can be suppressed, resulting in layer thicknesses of up to 3 nm for ZrBTB (Zr_6_(O)_4_(OH)_4_(OH)_6_(H_2_O)_6_(BTB)_2_ with BTB^3−^ = benzene-1,3,5-tribenzoate).^119^

## Applications

As discussed in the previous section, already a lot of effort has been made in the synthesis of two dimensional framework materials, giving rise to a handful of well-established systems, which can be produced as ultrathin sheets with enhanced access to catalytic sites, shortened diffusion pathways and unique optical properties. All of these properties, are interesting for a range of applications in the energy-sector, for instance in electrocatalysis, as electrode materials in supercapacitors or batteries, as integral parts of membranes for molecular sieving or for new state of the art optoelectronic devices. In the following we highlight the potential use of 2DFMs in these technologically highly relevant applications.

### Electrocatalysis

**Fig. 6:**
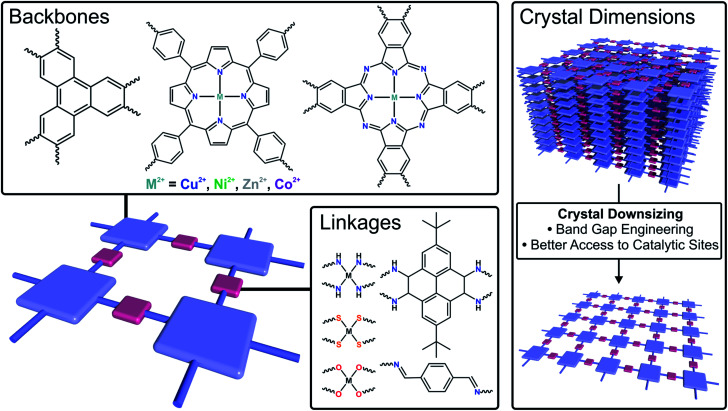
Schematic illustration of strategies to tune the electrocatalytic performance of 2DFMs by changing the backbone, the linkage or the crystal dimensions.

Currently, exploring advanced electrocatalysts has been the grand challenge of energy conversion technologies.^120,121^ Due to the precise tunability in composition/structure, high surface area, dense exposed catalytic sites and improved conductivity compared to their bulk counterparts, 2DFMs are highly promising electrocatalysts with high conversion efficiency at limited energy input (Fig. 6).^122–126^ In this section, 2D framework electrocatalysts are discussed which are exploited in several crucial energy conversion processes, such as hydrogen evolution reaction (HER), oxygen evolution reaction (ORR), oxygen reduction reaction (ORR) and carbon dioxide reduction reaction (CO_2_RR).

The electrocatalytic HER *via* water splitting has been widely regarded as a clean and sustainable way for the production of high quality H_2_. Typically, integrating the molecular transition metal (Fe, Co, Ni, *etc.*) complexes into 2D scaffolds has been explored to boost the HER catalyst activity of 2D framework catalysts.^100,101,127–130^ For instance, a cobalt dithiolene fused 2D MOF (MOS) was successfully synthesized *via* a liquid–liquid interfacial process by the Marinescu group.^127^ When applied in the electrocatalytic HER, the target MOS electrocatalyst displayed a good HER performance with an overpotential of 0.3 V at 10 mA cm^−2^ in acidic media, owing to the high loading with catalytic sites (CoS_4_) and remarkable stability. Subsequently, various metal dithiolene based 2D frameworks electrocatalysts were developed for the electrocatalytic HER.^101^ Apart from metal dithiolene sites, Dong *et al.* rationally incorporated metal dithiolene–diamine (MS_2_N_2_, M = Co/Ni) into carbon-rich 2D MOFs as a model carbon electrocatalyst for HER. The electrocatalytic HER activity of these 2D MOFs varied for the different metal coordination modes in the sequence of MS_2_N_2_ > MN_4_ > MS_4_. It is illustrated that the protonation process prefers to occur on M–N sites located in the MS_2_N_2_ coordination spheres of the nanosheets. This work offered a comprehensive understanding of various metal complexes (MN_*x*_ and MS_*x*_) and shed light on the design of high-performance catalyst. Besides, metal-free 2D COFs with abundant functional groups were also synthesized for electrocatalytic HER. The Pradhan group first reported an imine-based conjugated pyrene and porphyrin based 2D COF (SB-PORPy COF) as a metal-free HER electrocatalyst.^132^ The SB-PORPy COF exhibited a remarkable catalytic activity with low onset potential of 50 mV and excellent stability in acid electrolyte, wherein the imine nitrogen sites are acting as catalytic centers. Despite significant progress, the catalytic activities of current 2D framework based electrocatalysts are still inferior to the benchmark inorganic catalysts, mostly due to the lower stability and overall conductivity. A summary of key properties of 2DFMs in the HER can be found in Table 1.

**Table 1:** Performance of different 2DFMs in the hydrogen evolution reaction (HER)

Material	Type	Thickness (nm)	Onset potential (V *vs.* RHE)	Overpotential^a^ (mV)	Tafel slope (mV dec^−1^)	Exchange current density (×10^−4^ A cm^−2^)	Reference
Co_3_(BHT)_2_	MOF	360	−0.28 *vs.* SHE	340	149	10^−1.2^	127
THTNi 2DSP	MOF	0.7	−0.11	330	80.5	6	100
2D CTGU-5	MOF		−0.298	388	125	8.6	131
SB-PORPy COF	COF	80–150	−0.05	380/5 mA cm^−2^	116	—	132
TpPAM	COF			250	106	2.4	133
2DCCOF1	COF	7.5		541	130	—	134

a Current density = 10 mA cm^−2^.

In contrast, for oxygen evolution 2D frameworks have shown remarkable performance comparable to the state-of-art OER electrocatalysts.^135–137,142–150^ Using a bottom-up approach, the Du group synthesized a nickel phthalocyanine-based 2D MOF (NiPc-MOF),^135^ which delivered an outstanding OER performance with low onset potential of 1.48 V and high mass activity of 883 A g^−1^ in alkaline media. It is emphasized that the two dimensional structure and good conductivity of 2D MOFs contributed greatly to the excellent OER activity. Ultrathin NiCo bimetallic framework nanosheets developed by Tang and coworkers required an extremely low overpotential of 250 mV at 10 mA cm^−2^ in OER in alkaline conditions,^136^ which is superior to the benchmark noble metal-based catalyst (IrO_2_ and RuO_2_). The results indicate, that the coordinatively unsaturated metal atoms and the coupling effect between Ni and Co metals were crucial for the high electrocatalytic activity. Other strategies include the fabrication of ultrathin nanosheet arrays or lattice-strained nanosheet arrays of 2D MOFs on current collectors and the application of hybrid 2D MOFs, *etc.* were also proposed to enhance the OER catalytic activities of MOFs.^142,148,149^ The performance of a range of different 2DFMs in the oxygen evolution reaction has been summarized in Table 2.

**Table 2:** Performance of different 2DFMs in the oxygen evolution reaction (OER)

Material	Type	Thickness (nm)	Onset potential (V *vs.* RHE)	Overpotential^a^ (mV)	Tafel slope (mV dec^−1^)	TOF/potential (s^−1^/V *vs.* RHE)	Ref.
NiPC-MOF	MOF	100–200	1.48	250	74	0.2/1.65	135
NiCo-UMOFN	MOF		1.39	189	42	0.86/0.3	136
Co-ZIF-9(iii)	MOF	2–4	1.61	380	55	0.2/1.65	137
Fe:2D-Co-NS@Ni	MOF	2	—	210	46	30/1.53	138
CoBDC-Fc0.17	MOF		—	178	61	0.034/1.47	139
C4-SHz COF	COF		1.47	320	39	—	140
COF-C4N	COF	—	—	349	64	—	141
Co-TpBpy	COF	—	—	400/1 mA cm^−2^	59	0.23	142

a Current density = 10 mA cm^−2^.

Moreover, layered frameworks also show promising progresses in promoting electrocatalytic ORR, even though the most pertinent examples only discuss their non-exfoliated versions.^121,151–154^ In 2016, the group of Dincǎ reported thin films of the conductive layered MOF Ni_3_(HITP)_2_ (thickness ∼120 nm, deposited on glassy carbon electrodes, HITP = 2,3,6,7,10,11-hexaiminotriphenylene), with well-defined NiN_4_ units as an ORR electrocatalyst in alkaline electrolyte.^151^ It shows a good onset potential of 0.82 V *vs.* RHE (reversible hydrogen electrode) in 0.1 M KOH electrolyte, and good stability during extended polarization, highlighting layered conductive MOFs as a powerful platform to develop tuneable and rational electrocatalysts. 2D MOFs formed by the use of different ratios of Ni/Co metal source with the HITP ligand (were reported by the Peng group).^153^ The Co_3_HITP_2_ showed a distorted quadrilateral configuration owing to the unpaired 3d_*z*_^2^ electron in Co atom and a decrease in conductivity. However, it exhibited a remarkable ORR performance (onset potential of 0.9 V *vs.* RHE and electron transfer number of 3.97) in alkaline electrolyte because the unpaired electron on Co 3d_*z*_^2^ in the catalytically active CoN_4_ centre is beneficial for promoting the binding of oxygen intermediates and thus accelerates the ORR energetics despite of the reduced electric conductivity. Contrary, Ni_3_HITP_2_ with weak binding of *OOH on NiN_4_ sites goes through a 2e^−^ pathway for ORR with lower catalytic activity. Therefore, optimizing the architecture and electronic structure of layered MOFs is an effective strategy to develop highly active electrocatalyst toward ORR. In Table 3 the key properties of some 2DFM based ORR catalysts have been summarized.

**Table 3:** Performance of different 2DFMs in the oxygen reduction reaction (ORR)

Material	Type	Thickness (nm)	Onset potential (V *vs.* RHE)	Halfwave potential (V *vs.* RHE)	Tafel slope (mV dec^−1^)	Transfer number	Reference
Ni_3_(HITP)_2_	MOF	120	0.82		128	2.25	151
Co_3_(HITP)_2_	MOF	2	0.79	0.80	112	3.96	153
Co_*x*_Ni_*y*_-CAT	MOF	—	0.46	0.34	104	3.94	164
PcCu-O_8_-Co	MOF		0.9	0.82	61	3.93	165
PTM-H-COF	COF	95	—	0.7	—	3.89	166
COP-PSO_3_-Co-rGO	COF	—	0.88		67.4	3.7/0.75 V	152

In CO_2_RR electrocatalysis, optimized 2D frameworks electrocatalysts would be an ideal choice to control the catalytic activity, selectivity and efficiency in a single catalytic system with high performance toward the target products.^155,156,159,160,167–169^ Ultrathin 2D MOF nanosheets with cobalt-porphyrins as active centers (TCPP(Co)/Zr-BTB) were developed for electrocatalytically transforming CO_2_RR to CO, showing high catalytic activity with a TOF of 4768 h^−1^ at −0.919 V *vs.* RHE. Besides, the post modification strategy was further applied to promote the catalytic performance. *p*-Sulfamidobenzoic acid modified samples exhibited an improved TOF of 5315 h^−1^ at −0.769 V *vs.* RHE along with a faradaic efficiency of 85.7% for CO and long-term durability.^155^ Therefore, high utilization of active sites and optimized post modification are effective strategies to enhance the CO_2_RR performance. For example, the Feng group developed a layer-stacked bimetallic conjugated MOF (2D c-MOFs), which is a highly active and selective electrocatalyst toward CO_2_RR.^156^ The optimized 2D c-MOF (PcCu-O_8_-Zn) displayed high CO selectivity of 88% and TOF of 0.39 s^−1^. A synergistic catalytic mechanism of the bimetallic 2D c-MOFs is proposed to contribute the catalytic performance, unravelled by *in situ* X-ray absorption spectroscopy, surface-enhanced infrared absorption spectroelectrochemistry, and theoretical calculation studies. There, ZnO_4_ complexes act as CO_2_RR catalytic sites while CuN_4_ centres promote the protonation of adsorbed CO_2_ during CO_2_RR, offering a new and effective strategy for developing MOF based electrocatalysts. Besides, the Lan group introduced oriented efficient electron transmission by metalloporphyrins, to achieve highly active electrocatalytic CO_2_ to CO conversion *via* the application of tetrathiafulvalene as an electron donator/carrier. The best candidate, Co-TTCOF, after exfoliation, exhibited a spectacular faradic efficiency for CO (more than 90% over a wider potential range), wherein the maximum value reaches up to 99.7%, which is superior to the current state-of-the-art catalysts.^160^ In Table 4 a summary of different CO_2_RR electrocatalysts can be found.

**Table 4:** Performance of different 2DFMs in the carbon dioxide reduction reaction (CO_2_RR)

Material	Type	Thickness (nm)	TOF (h^−1^)/potential (V *vs.* RHE)	Faradaic efficiency (%)/potential (V *vs.* RHE)	Tafel slope (mV dec^−1^)	Reference
TCPP(Co)/Zr-BTB	MOF	1.9	4768/−0.919 V	85.7	122	155
PcCu-O_8_-Zn	MOF	24	1404/−0.7	88	125	156
Ni(Im)_2_	MOF	5	770	78.8/−0.85	137	157
MOF-NS-Co	MOF	3.16 ± 0.23	11 762	98.7/−0.6	268	158
COF-366-Co	COF	350	9400/−0.55	90	470–550	159
Co-TTCOF	COF	5	4608/−0.7	99.7	237	160
TT-Por(Co)-COF	COF	1.3		91.4/−0.6		161
NiPc-TFPN COF	COF	—	490/−0.9	99.8(±1.24)/−0.9	209.9	162
CoPc-PDQ-COF	COF		11 412	96/−0.67	112	163

While reports on the electrocatalysis on 2DFMs remain scarce, the progress in the field of layered framework electrocatalysts (essentially the precursors for the underlying 2DFMs) is intriguing. This might motivate more groups to test two dimensional versions of high performance layered framework structures, to further promote the development of electrocatalysts for energy conversion processes.

### Batteries and supercapacitors

**Fig. 7:**
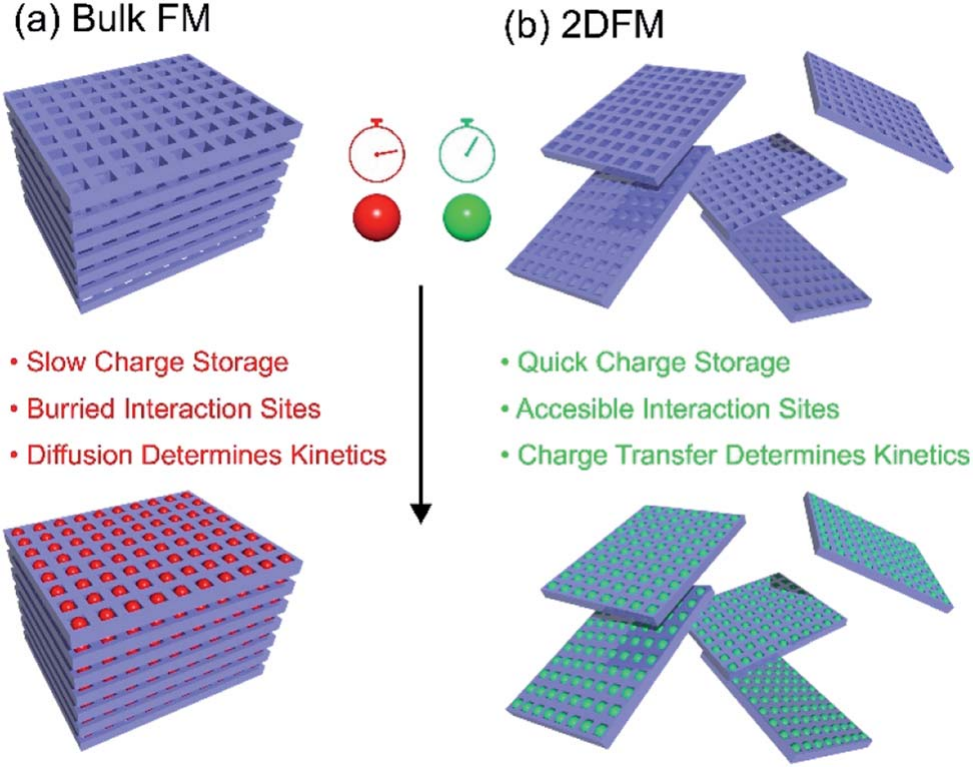
Schematic depiction of the charge storage processes in bulk framework materials and 2DFMs.

Rapid storage/release of energy, increased storage capacities and cycle life are currently among the key aspects in the development of new electrode materials for batteries and supercapacitors. In recent years, a lot of attention focused on the use of 2D materials as the active components in electrodes.^170–173^ This is because of the considerable increase of the surface-to-volume-ratio in a 2D exfoliated state in comparison to bulk, and hence the facilitated access to the redox active sites. Thus, a much more intimate contact between the electrode and the electrolyte occurs and the overall charge transport pathways through the material are considerably shortened. All these effects improve the charging/discharging kinetics (*i.e.* diffusion controlled kinetics *vs.* redox reaction controlled kinetics), which in turn have a major influence on the delivered specific capacity of the device.^174^ The use of porous 2D framework materials gives the additional advantage that redox-active groups can be installed within the pore space of the electrode material, and in contrast to bulk framework materials or polymers with redox active groups, these redox active sites will not be buried deep inside the crystalline/polymer structure.^175^ On the other hand for practical applications a rather critical aspect often overlooked is the increased dead volume of highly porous card house structures leading to a high contribution of inactive mass (electrolyte). The bare material mass-related specific energy (or power density) does not take this into account and for real world applications packing density is a very important parameter for energy density optimization at stack level. Moreover, in particular for batteries the high irreversible loss due to Solid Electrolyte Interphase (SEI) formation is a critical aspect of 2D materials. Fig. 7 gives an overview of some advantages of 2DFMs in applications in batteries and supercapacitors.

In the following, we are giving some examples on the use of 2DFMs and how the targeted preparation of 2DFMs can improve charge and ion storage and delivery compared to their bulk counterparts. 2D MOF and COF-derived materials for energy applications are a widely discussed topic, but this huge field is out of the scope of this article and is discussed elsewhere.^176–179^

Since 2DFMs combine both the positive properties of 2D materials and the advantages of porous framework compounds, resulting in high modularity, exceptionally large surface area and a high surface-to-volume ratio, they fulfil many requirements for outstanding energy storage materials. The performance of different 2DFMs in battery and supercapacitor applications have been summarized in Tables 5 and 6, respectively.

**Table 5:** Summary of the performance of different 2DFMS used as electrode materials for lithium ion batteries (LIB), lithium–air, lithium organic and sodium ion batteries (SIB)

Material	Thickness (nm)	Type	Specific capacity (mA h g^−1^)	Current density (mA g^−1^)	Cycles	Li^+^ diffusion coefficient (cm^2^ s^−1^)	Ref.
E-TFPB-COF/MnO_2_	1.6–2.0	LIB	1359	100	300		180
(IISERP)-CON1	2–4	LIB	720	100	1000	5.48 × 10^−11^	181
(IISERP)-CON2	0.8–1.5	LIB	790	100	1000	3.69 × 10^−11^	85
DAAQ-ECOF	3–5	LIB	107	500	1800	6.94 × 10^−11^	82
PI-ECPF-1/rGO50	—	LIB	∼115	1000	300		182
E-FCTF	4.2	LIB	1035	100	300	2.36 × 10^−10^ to 7.88 × 10^−9^	83
f-CTF	1.2–1.9	LIB	560–650	1000	500	3.75 × 10^−13^	76
Mn-UMOFNs	—	LIB	1187	100	100	2.48 × 10^−9^	183
u-CoTDA	1–10	LIB	790	1000	400		184
Mn-MOF	5.3	Li–air	9464	100	200		185
E-CIN-1/CNT	2	Li–organic	744	100	250	3.29 × 10^−19^ to 5.84 × 10^−18^	81
CON-16	—	SIB	250	100	30	NA	186
CTF-1	2–3	SIB	266	100	60	NA	187

**Table 6:** Performance of different 2DFMs tested as electrode materials in supercapacitors

Material	Type	Thickness	Capacitance	Current density	Cycling stability	Reference
NiCoMOF	MOF	3.1 nm	1038 F g^−1^	5 A g^−1^	89.5% over 5000 cycles	188
JUC-511	COF	22 nm	5.46 mF cm^−2^	1 A g^−1^	∼100% over 1000 cycles	192
Co-MOF	MOF	2 nm	1106 F g^−1^	2 A g^−1^	96.7% over 6000 cycles	189
NiCO-MOF/rGO	MOF	—	1553 F g^−1^	1 A g^−1^	83.6% over 5000 cycles	190
Ni-TCPP nanofilm/CNT	MOF	2 nm	2280 F g^−1^	5A g^−1^	90.3% over 2000 cycles	191
Ni_3_(HTTP)_2_	MOF	—	∼100 F g^−1^	2 A g^−1^	90% over 10 000 cycles	193

The groups around Yang and Pang have synthesized ultra-thin MOF nanolayers (2–3 nm) based on the 1,4-benzenedicarboxylate (bdc^2−^) linker.^188,189^ Thereby comparable specific capacities could be achieved for Co^189^ and a mixture of Ni and Co^188^ of 1159 F g^−1^ and 1202 F g^−1^, respectively, with high cycle stability. Additionally, Yang and co-workers present an asymmetric supercapacitor of NiCo-MOF//activated carbon composite which delivers an energy density of 49.4 W h kg^−1^ at a power density of 562.5 W kg^−1^ in a voltage window in the range from 0–1.5 V.^188^ Furthermore, the use of hybrid materials based on 2D MOFs is a well-established strategy to improve poor electrochemical performance of pure MOFs caused by the low conductivity of many MOFs. Thereby it is irrelevant whether the conductive additives are introduced post-synthetically or *in situ* during the synthesis. The latter was favoured by Beka *et al.*^190^ to deposit a 2-methylimidazole NiCo-2D MOF on rGO which results in a specific capacitance of 1553 F g^−1^ at a current density of 1 A g^−1^. In addition, the assembled asymmetric device showed an excellent energy density of 44 W h kg^−1^ at a power density of 3168 W kg^−1^. Bai *et al.*^191^ presented various M-TCPP 2D MOFs, (M^2+^ = Cu^2+^, Co^2+^ and Ni^2+^, TCPP^4−^ = tetrakis(4-carboxyphenyl)porphyrin) and their corresponding composites with CNTs and GO. In supercapacitor applications, Ni-TCPP (2 nm thick) on CNT exhibited a large specific capacitance of 2280 F g^−1^ at a current density of 5 A g^−1^ and 1450 F g^−1^ even at 20 A g^−1^, which shows its potential for high power applications. Yusran and coworkers exfoliated an isoreticular series of porphyrin based imine linked mesoporous COFs. Namely, JUC-510, JUC-511 and JUC-512, which contain no metal species at the porphyrin center, Ni^2+^ and Co^2+^, respectively. The nickel containing JUC-511 could be exfoliated into 22 nm thick sheets with an areal capacitance of 5.46 mF cm^−2^ retaining nearly 100% of it over the course of 1000 cycles. Further, a high power density of 55 kW kg^−1^ was found.

Due to the very simple exfoliation methods, the convenient availability and the easy handling, 2D MOFs, based on the H_2_bdc linker, are also widely utilized in various battery applications.^183,185,194^ Especially the manganese compound could demonstrate better results than their cobalt and nickel derivatives both as anode material for LIBs^183^ and as cathode material in aprotic Li–O_2_ batteries.^185^ In addition, Li *et al.*^194^ designed a separator based on ultrathin (1.2 nm) Co_2_(bdc)_2_ nanosheets which enhances the safety and lifetime of lithium–sulphur batteries by simultaneously suppressing Li dendrite growth and alleviating polysulfide shuttling. The periodically arranged cobalt atoms coordinated with oxygen atoms (Co–O_4_ moieties) exposed on the surface of the ultrathin MOF nanosheets, can greatly homogenize the Li ion flux through the strong Li ion adsorption to the O atoms at the interface between anode and separator, leading to stable Li striping/plating. Meanwhile, the well accessible cobalt atoms of the 2D MOF sheets serve as “traps” to suppress polysulfide shuttling by Lewis acid–base interaction at the cathode, resulting in a bifunctional separator. As a result, the Li–S coin cell exhibits a long cycle life with an ultralow capacity decay of 0.07% per cycle over 600 cycles. Even with a high sulphur loading of 7.8 mg cm^−2^ and an areal capacity of 5.0 mA h cm^−2^ after 200 cycles. In 2017, Hu's group was already able to demonstrate the advantages of 2D materials compared to their bulk counterparts and that both the metal centre and the linker have a major influence on the oxidation and reduction processes during lithium ion storage. A 2D MOF with a thickness of up to 10 nm, consisting of Co and thiophenedicarboxylate, exhibits a high reversible capacity (790 mA h g^−1^ after 400 cycles at 1 A g^−1^) and excellent rate capability (694 mA h g^−1^ at 2 A g^−1^) as an anode material in Li-ion coin cells.^184^ Wang and co-workers exfoliated an imine-linked COF that features redox active anthraquinone moieties on the linker backbone into 5 nm thick sheets. These COFs were tested as cathode materials for lithium ion batteries. Contrary to the bulk material, the redox reactions in 2D COF are controlled by charge transfer, because of the reduced diffusion pathways. The material delivered 96% of its theoretical capacity at 20 mA g^−1^ and retained even after 1800 cycles a capacity of 104 mA h g^−1^ at 500 mA g^−1^, demonstrating the utility of nanosizing redox active 2DFMs.^82^ Haldar and co-workers exfoliated an anthracene based imine-linked COF through a Diels Alder reaction with maleic anhydride, thus creating redox active sites on the attached groups. The material additionally featured keto-groups on the tritopic linker that bridge the anthracenes.^85^ Interestingly, the few layer thick material had a fourfold increase in specific capacity compared to the bulk counterpart. Incorporation into a realistic fuel cell type set up using LiCoO_2_ as a cathode material could retain a specific capacity of 220 mA g^−1^ over 200 cycles, placing it highest among all organic polymer material based anodes.

Further progress was made in the field of Ni–Zn and Zn–air batteries through the application of composite materials.^195,196^ Li *et al.* demonstrated the *in situ* deposition of a nickel based 2,6-naphthalene dicarboxylate material on highly flexible CNTs in an Ni–Zn battery resulting in a high areal capacity (0.4 mA h cm^−2^ at 0.5 mA cm^−2^), remarkable energy density of 0.71 mW h cm^−2^ and great rate capacity after cycling 600 times.^195^ The group around Zhong focused on the use of nanolayers of a bimetallic TCPP MOF compound on rGO. Using CoNi-MOF/rGO as the air electrode, the rechargeable Zn–air battery was assembled and displayed a stable open circuit voltage, excellent energy density, and long-time cycling stability.^196^

### Optoelectronics

The transduction of electrical-to-optical signals or *vice versa*, is the working principle of technologically important optoelectronic devices, such as photodiodes, light emitting diodes, photoresistors or photovoltaics. In general, the optical properties of 2DMs depend on the number of layers in the resulting materials structure.^197^ The band structure of the applied materials is usually responsible for the underlying properties, and in classical 2DMs interesting changes in the band gap are observed, when downsizing to atomistic thickness occurs along the stacking direction. For instance, MoS_2_ features an indirect bandgap in its bulk layered state which transforms to a direct bandgap upon nanosizing along the third dimension.^198^ For 2D framework materials similar implications are expected and when build up from organic fluorophores, photoelectron transfer (PET) between adjacent layers can be inhibited by the elimination of π–π stacking upon exfoliation. Furthermore, the conjugation within the layers can be widely adjusted by choice of the linkage between the accessible building blocks allowing for precise tuning. The pore space furthermore gives rise to the incorporation of additional antennas or photosensitizers within the layers or might offer binding/accumulation sites for analytes in optical sensing applications.

The optical properties of the 3D analogues of the discussed framework materials are well explored, since the incorporation of fluorophores into frameworks, inhibits their aggregation and hence undesirable effects like PET.^199–201^ However, layered materials with short interlayer-distances might be excluded from this blanket statement. In the case of layered COFs, many of the early materials where assembled from organic fluorophores.^61^ The use of bandgap engineering to systematically tune the optical properties is currently mostly achieved by targeted combination of the frameworks components,^202–204^ although some studies describe the use of nanoscaling along the third dimension as it is described for more traditional 2D materials, which will be discussed in the following.

Probably the most widely researched optoelectronic application of 2DFMs is their use as sensors, *i.e.* for metal ions, pollutants or biomolecules and is based on the change of their optical properties upon interaction with the analyte. A study by the group of Zhao, for instance, shows how the exfoliation of layered azine-linked COFs to 2–4 nm thick 2D COFs enhances the detection of amino acids.^205^ Compared to the bulk material, a stronger fluorescence quenching is observed and hence a stronger signal. The authors link these observations to the inhibition of aggregation caused quenching and easier access to binding sites on the surface of the COFs. In another study, Peng and co-workers used 2D COFs for the detection of DNA. In their study, an imine-bridged COF was delaminated by liquid-assisted exfoliation into ultrathin nanosheets (3.5 nm).^228^

2DFMs offer many properties, which can be helpful for the use in photovoltaics. If built up from optically transparent, fluorophores, they can be potential light absorbers and can generate and transport carriers and additionally their flat extended shape helps aligning them in device configurations. In comparison to light harvesting organic polymers, the two-dimensional framework structure offers a long-range order, which minimize the presence of traps, dead ends and defects which hamper the light harvesting efficiency. The group of Foster used liquid-assisted exfoliation to prepare zinc-porphyrin based 2D MOFs and integrated them into a polythiophene–fullerene (P3HT–PCBM) organic solar cell set-up.^206^ The incorporation into the organic photovoltaic led to a doubling of the devices performance. The 2D MOFs bandgap is intermediate of the acceptor and donor of the system and hence does not create traps for charges. The authors suggest that the marked increase in open circuit voltage, current density and fill factor of the prepared solar cells derives from the 2D MOF nanosheets acting as a template that enhances the crystallinity of the P3HT and prevents PCBM over-growth. Park and co-workers illustrated in a study the use of a 2D COF prepared by the Stille coupling as a hole transport layer in perovskite solar cells.^207^ In device arrangements featuring the 2D COF, an increase in power conversion efficiency of 1% is observed. Generally speaking, the use of 2DFMs in photovoltaics are still only scarcely researched, even though plenty of studies on 3D and layered framework materials exist, still there is a long way to go to reach the full potential of these materials.^208–210^

Jiang and co-workers prepared the layered MOF material [Ni_3_(OH)_2_(bdc)_4_],^211^ which readily exfoliates to 4.2 nm sheets upon inclusion into a saturable absorber precursor solution.^212^ The preparation process also lead to the inclusion of Ni ions into the framework nanosheets, which lead to a striking shift of the bandgap from 3.12 eV (bulk, no Ni doping) to 0.86 eV, comparable to conventional 2D materials. Interestingly, this material revealed optical amplitude modulation properties, and implementation of the MOF-based saturable absorber into a fibre resonator demonstrated its usability for mode locking operation in the telecommunication wavelength window.

Similarly, the group of Lin looked at the white light emission of bilayers of a Zr_6_O_4_ based MOF with a tetratopic tetraphenylethylene-based linker.^213^ This material showed three times higher physical switching speed compared to commercial white-light emitting diodes, which is an interesting feature for application in visible-light based communication.

Ding and co-workers prepared Zn-porphyrin based 2D MOF with an average thickness of ∼8 layers through a surfactant assisted synthesis route.^214^ The ultrathin nanosheets were incorporated into a resistive random access memory device as the resistive layer and showed excellent reliability and resistive switching properties compared to other resistive switching devices.

Mukhopadhyay and co-workers exfoliated 2D MOFs consisting of chromophoric linkers with photoswitchable units and incorporated the 2–5 layer thick nanosheets into an ormosil polymer matrix.^215^ Interestingly, the intrinsic void volume of the nanosheets accounted for the volume change of the photoswitch. Furthermore, the preparation of nanosheets lead to a homogeneous distribution of the MOFs in the ormosil matrix. The material shows T-type photochromism, turns green upon irradiation and bleaches again after termination of the stimulus. The composite is stable towards several colorization/decolorization cycles and shows the potential of 2DFMs for use in advanced lenses and for technological glasses.

Apart from sensing applications, the use of 2DFMs in optoelectronic applications is still in its infancy, even though this class of materials holds a lot of promise for their employment as constituents of novel electronic components, particularly through the potential of tuning the electronic properties and band structure by simple exchange of building blocks.^216,217^

### Membrane technology

**Fig. 8:**
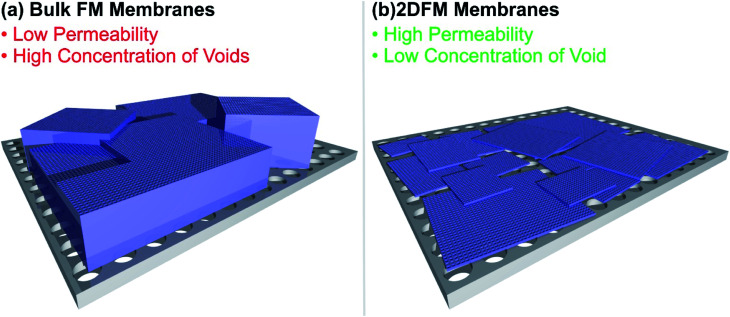
Illustration of the difference of a bulk framework material membrane and a 2D framework material membrane placed on a porous substrate.

Selectivity and permeability are the two most important properties of separation membranes. The permeability describes the flux of molecules through the membrane and ultimately determines the throughput, and the selectivity describes the ability of a component to pass through the membrane in comparison to another constituent of a mixture. In comparison to other separation techniques, membranes offer a lot of promising merits, including high energy efficiency, economic viability and easy scale up. Conventional membranes, which traditionally consist of dense and amorphous polymers, usually show a trade-off between permeability and selectivity. The use of mixed-matrix membranes featuring porous materials such as MOFs, COFs or 2D polymers as fillers triggered a lot of promising research, creating materials with high selectivities for molecular separations and good flux.^218–225^ However, the interfacial incompatibility between the porous filler and the polymer often leads to the formation of voids and pinholes, which create non-selective pathways through the membrane. The construction of defect-free membranes with framework material based fillers has been demonstrated, although relatively thick fillers were used, in the range of tens of micrometres. This poses another problem, since increasingly thick separation fillers are hampering the mass transport through the membrane and are an issue for the permeation flux. Furthermore, the use of 2D framework materials with large lateral extensions would lead to a better distribution of the filler over the membrane cross section and consequently to a decreased amount of cracks and undesirable flux pathways in comparison to isotropic crystals. The use of defect free, free-standing 2DFM monolayers would be an idealized approach to overcome the trade-off between selectivity and permeability, however the preparation of large area free-standing monolayer membranes is not practical and rarely achieved. The mechanical stability of such monolayer membranes is a limiting factor (see Fig. 8 for a schematic). Currently, the use of ultrathin 2DFMs as fillers or as laminated layers placed on porous supports^226^ offers an attractive route to high flux/high selectivity membranes and in the following discuss recent progress and give a future perspective.

One of the most notable examples for the use of 2DFMs as filler materials was presented by Rodenas *et al.* In their study, 30–500 nm thick Cu_2_(bdc)_2_ nanosheets with micrometre sized lateral extensions were incorporated into a polyimide matrix. In comparison to control experiment with bulk crystallites, the 2D filler distribution over the membrane is much more homogeneous. Interestingly, the fabrication of such mixed matrix membranes (MMMs) filled with nanosheets also leads to an increase in selectivity towards CO_2_ over CH_4_ in comparison to bulk. Furthermore, with an increase in applied pressure, the selectivity increases, while for the bulk material, the selectivity significantly drops when a *trans*-membrane pressure of Δ*p* = 5 bar is surpassed. In another study, the group of Zhao exfoliated the layered MOF Ni_8_(5-bbdc)_6_(OH)_4_ (5-bbdc^2−^ = 5-*tert*-butyl-1,3-benzenedicarboxylate) by freeze–thaw exfoliation to thicknesses as low as 4 nm with very high aspect ratios.^227^ The nanosheets were aligned on anodic aluminium oxide supports by hot dropcasting from DMSO. The membranes show very high selectivity towards H_2_ over CO_2_ (separation factor of 245). The pores are lined with *tert*-butyl groups, which lead to a contraction of the pores at enhanced temperatures, which significantly increases the selectivity. In another study, the group of Zhao assembled heterolayers of anionic and cationic COFs on top of a porous α-Al_2_O_3_ support.^74^ The COFs were assembled *via* the Langmuir–Schäfer method by alternatingly dipping the support into suspensions of the ionic COFs. In this fashion, the thickness of the layers could be controlled. Through the assembly of the anionic and cationic COFs, which are stacked in a staggered fashion, the effective pore diameter is reduced. The hetero-stack of COF nanosheets shows a selectivity towards H_2_ over CO_2_ of 25.2 at 423 K, which is considerably larger than the selectivity of the individual components.

Currently, a lot of effort is focussed on the preparation of 2DFM material based membranes. The combination of porous supports with 2D materials with large lateral extensions seems very promising, although, the preparation of micrometer or even millimetre sized stable selective single layers has not been achieved yet, clever ways to place delaminated sheets in a targeted fashion on such supports are being developed. Furthermore, the preparation of MMMs featuring 2DFMs as filler is also a promising technique to obtain highly selective membranes with good permeabilities. In case of separation problems that are being tackled, it would be desirable if other, hard to achieve separations could be targeted, for instance the separation of short chained hydrocarbons.

## Conclusions

The development of 2D framework materials experiences a highly dynamic growth, with rapid improvements in the development of new exfoliation methods paving the way towards different technological applications in the energies sector. Currently, it is necessary that the community decides on a common nomenclature and some guidelines, which ensures that all researchers are on the same page. Right now, there is a lot of confusion in the literature, as researchers describe with the term 2D COFs and 2D MOFs on the one hand ultrathin 2D materials, but also bulk layered sheet structures. Introducing the terms MOFene or COFene for such monolayers would lead to an intuitive clarification acceptable for textbook chemistry. Furthermore, a lot of research that discusses the effect of nanosizing along one axis on materials properties, usually only describes one ultrathin material, which is often not properly characterized by appropriate methods (*i.e.* microscopy). In order to gain further understanding on the underlying effects, researchers should take an aim to widen the amount of materials compared and not only compare a bulk material with a single undefined exfoliated material. Furthermore, a lot of research that is conducted is limited to the behaviour of a few rather well understood materials systems, opening interesting opportunities to explore applicability of concepts discovered so far, to more complex systems tailored towards application. Within the field of 2DFMs, also always a close look to developments in the area of conventional 2DMs should be taken. The determination of physical properties like charge transfer kinetics, surface energy, flexibility of sheets is currently lacking in the literature. The further development of easy access methods outside of the traditional microscopy techniques to determine actual thicknesses of such 2DFMs is also critical. Finally, a lot of promising application arise through the compatibility with many device configuration that allow the integration of flat two dimensional sheets. Despite material specific quantities of compounds with low packing density should not be overrated, a lot of very promising research towards energy related application is already being conducted, and we expect, that with a greater understanding and control of the formation of few layer structures, even better results can be obtained.

## Conflicts of interest

There are no conflicts to declare.

## Supplementary Material
